# Acute Effects of Esports on the Cardiovascular System and Energy Expenditure in Amateur Esports Players

**DOI:** 10.3389/fspor.2022.824006

**Published:** 2022-03-11

**Authors:** Rebecca T. Zimmer, Sandra Haupt, Heiko Heidenreich, Walter F. J. Schmidt

**Affiliations:** ^1^Division of Exercise Physiology and Metabolism, Department of Sport Science, University of Bayreuth, Bayreuth, Germany; ^2^Division of Sport Governance and Event Management, Department of Sport Science, University of Bayreuth, Bayreuth, Germany; ^3^Department of Sport Science, University of Bayreuth, Bayreuth, Germany

**Keywords:** gaming, stress, sympathetic system, cortisol, heart rate, oxygen uptake

## Abstract

**Introduction:**

Esports is practiced by millions of people worldwide every day. On a professional level, esports has been proven to have a high stress potential and is sometimes considered equivalent to traditional sporting activities. While traditional sports have health-promoting effects through muscle activity and increased energy expenditure, amateur esports could represent a purely sedentary activity, which would carry potentially harmful effects when practiced regularly. Therefore, this study aims to investigate the acute effects of esports on the cardiovascular system and energy expenditure in amateur esports players to show whether esports can be considered as physical strain or mental stress or whether amateur esports has to be seen as purely sedentary behavior.

**Methods:**

Thirty male subjects participated in a 30-min gaming session, playing the soccer simulation game FIFA 20 or the tactical, first-person multiplayer shooter Counter-Strike: Global Offensive. Respiratory and cardiovascular parameters, as well as energy expenditure, blood glucose, lactate, and cortisol, were determined pre-, during, and post-gaming.

**Results:**

There were no significant changes in oxygen uptake, carbon dioxide output, energy expenditure, stroke volume, or lactate levels. Heart rate, blood glucose and cortisol decreased through the intervention until reaching their minimum levels 10 min post-gaming (Cortisol_pre_: 3.1 ± 2.9 ng/ml, Cortisol_post_: 2.2 ± 2.3 ng/ml, *p* < 0.01; HR_min0.5_: 82 ± 11 bpm, HR_post_: 74 ± 13 bpm, *p* < 0.01).

**Conclusion:**

A 30-min esports intervention does not positively affect energy expenditure or metabolism in amateur esports players. Therefore, it cannot provide the same health-promoting effects as traditional sports participation, but could in the long-term rather cause the same potentially health-damaging effects as purely sedentary behavior. However, it does not trigger a negative stress response in the players. Deliberate physical activity and exercise routines adapted to these demands should therefore be part of the daily life of amateur esports players.

## Introduction

Esports at different performance levels, defined as a competitive sport where gamers use their physical and mental abilities to compete in various games in a virtual, electronic environment (International Esports Federation, 2021), have grown tremendously in recent years. For example, the popular esports game League of Legends was played by an average of over 27 million people every day in 2018 (Newzoo, 2018). The total number of videogame players is significantly higher due to the variety of games and is further boosted by the restrictions on sports and activities during the COVID-19 pandemic (Goodwin, 2020). Since there are no hard differentiation criteria in the literature so far, for this study, we distinguish three performance levels of videogame players: On one side, professional esports players who have developed their game-specific physical and mental skills to the highest degree in order to establish themselves at the elite level of organized esports and often practice 8–12 h a day (Khromov et al., 2018). On the other side, casual gamers should be distinguished from esports players, as they do not require the prolonged development of skills in one game as well as competitive gaming (Reitman et al., 2020). However, casual gamers often also spend several hours a day playing various games (Khromov et al., 2018). As a third, intermediate performance level, we consider amateur players who focus on developing skills in a specific game and play this game in a competitive setting but have not reached a professional level (Jagnow, 2018; Khromov et al., 2019).

This trial will focus on amateur esports players since professional esports players represent only a small part of the total esports player population. For example, there were ~202 million video gamers (all skill levels) in the U.S. in 2020 (Statista Research Department, 2021), of which 4,334 played professionally (Gough, 2021). Due to the lack of differentiation criteria, no solid numbers for amateur players can be given. However, due to the high numbers of video gamers in general and the fact that only a few of many aspiring amateur gamers make it to the professional level, it can be assumed that amateur players represent a correspondingly large cohort. Since this population group puts a high amount of time into competitive esports, the amateur sector is highly relevant from an epidemiological point of view. Overall, esports is steadily growing in importance and must therefore be viewed as a widespread social phenomenon with potential medical consequences.

Although esports is controversially discussed at the highest economic, political, and sports policy levels (Jenny et al., 2017; Ansgar and Jannika, 2018; Fiore et al., 2020), it is gaining acceptance in the world of athletics (Holden et al., 2017). However, today, the health-promoting aspect is mainly associated with traditional sports: systematic endurance training improves cardiovascular health, and regular muscle contractions during endurance or strength training release a large number of myokines, which have proven to be very positive in the prevention of many diseases of civilization (Wilmore, 2003; Chen et al., 2016). Whether these preventive effects are also caused by esports is questionable, as it is still unclear whether esports is a purely sedentary task or whether it causes metabolic activity in the players. Following, we assume that esports is a purely sedentary activity if no change in energy expenditure (EE) of +14% is achieved. We base this assumption on the meta-analysis by Saeidifard et al. from 2018, who found a change in EE between purely sedentary and passive standing behavior of approximately +14% across 1,184 subjects (Saeidifard et al., 2018). This is supported by Amaro-Gahete et al. in 2019, who also measured a difference of approximately +14% between sitting and standing in *n* = 15 men (Amaro-Gahete et al., 2019).

Since the player often spends several hours a day in a primarily sedentary position and hardly any larger muscle groups are used, the potentially harmful effects of sedentary behavior in general could play a major role in the long-term. Greater time spent in a sedentary position is associated, for example, with higher all-cause mortality rates as well as increased risks for cardiovascular diseases and type 2 diabetes (Katzmarzyk et al., 2019; Saunders et al., 2020). In addition, a graded dose-response relationship between greater sedentary behavior and higher levels of different indicators of weight status, like body weight, body fat or adiposity, is assumed (Katzmarzyk et al., 2019; Saunders et al., 2020). Moreover, the harmful effects of sedentary behavior are more pronounced in physically inactive people (Katzmarzyk et al., 2019).

Applying the above findings to esports, there arise more and more health concerns because of the sedentary nature of esports and accompanying poor posture. It has already been observed that esports players are more likely to have musculoskeletal injuries like dysfunctions of the cervical and lumbar spine and the upper extremity (Zwibel et al., 2019); additionally, metabolic disturbances resulting from light-emitting diode computer monitors and mental health concerns regarding gaming addiction and social behavior disorders have been reported (Zwibel et al., 2019).

To this day, there are almost no data concerning metabolic changes caused by esports, and little research exists on cardiovascular stress. To answer the health impact question of esports, some studies regarding physiological stress in esports were carried out. These refer to either professional esports in a tournament situation or casual gaming (CG) (Staude-Müller et al., 2008; Lyons et al., 2011; Siervo et al., 2013, 2018; Rudolf et al., 2016; Behnke et al., 2019). In the competitive esports setting, significant increases in cardiac output ($\overset{\cdot }{\text{Q}}$) (Behnke et al., 2019) and heart rate (HR) (Chaput et al., 2011; Behnke et al., 2019) were measured. Since, in some cases, HR of up to 160–180 bpm were achieved and similar levels of cortisol as racing drivers were reached, there is a prevailing opinion that the stress load of professional esports players is comparable to traditional athletes (Rudolf et al., 2016; Schütz, 2016).

Looking at non-competitive CG, the data representing the stress reaction are inconsistent, showing slight increases, unchanged values or decreases for heart rate and unchanged or decreasing values for blood pressure and cortisol (Ballard and Wiest, 1996; Ballard et al., 2006; Arriaga et al., 2008; Barlett et al., 2008, 2009; Adachi and Willoughby, 2011; Siervo et al., 2013, 2018; Yeo et al., 2017). Overall, the available data tend to show that there are no or only minor stress reactions in CG. A similar inconsistency was seen in metabolic parameters, where the existing studies on CG found no or only a slight increase in oxygen uptake ($\overset{\cdot }{\text{V}}$O_2_) and EE (Lanningham-Foster et al., 2009; Lyons et al., 2011; Barry et al., 2016). At the same time, the respiration rate in the non-competitive setting decreased during the game (Staude-Müller et al., 2008). Thus, in CG, there appears to be no metabolic response and thereby no increase in EE. This would classify CG as purely sedentary behavior and CG could thus carry the possible long-term health risks of prolonged sedentary behavior. Overall, it turns out that an important distinction needs to be made between CG and professional esports, as physiological responses here are different and not transferable.

As previously described, there already exist studies on stress and metabolic responses in CG and, to some extent, professional esports settings. These studies, however, segmented gamers into a binary distinction between professional players and casual gamers. The intermediate performance level of amateur esports players has thus far not been considered, and so there is almost no research on how and to what extent amateur esports affect metabolism and EE and whether this is coupled with the stress responses. The only available study linking metabolic and stress data in amateur esports demonstrated that the stress response is decoupled from the metabolic response as HR was markedly increased while EE did not change (Haupt et al., 2021). However, this case study has weak explanatory power, and further research is urgently required. Furthermore, the barely studied segment of amateur esports players plays a major role from a health perspective due to the high number of players, especially young players, and needs to be investigated (Khromov et al., 2018; Hedlund, 2021).

This study aims to investigate the acute effects of esports on the cardiovascular system and EE in amateur esports players to show whether amateur esports has to be seen as purely sedentary behavior, possibly accompanied by potentially harmful effects on health in the long term, or whether esports can be considered physical strain or mental stress. To test this hypothesis, we focus our study on the two esports titles “Counter-Strike: Global Offensive” and “FIFA 20.”

## Materials and Methods

All investigations were conducted in accordance with the “Declaration of Helsinki” on Ethical Principles for Medical Research on Humans (World Medical Association, 2013), and the test protocol was approved by the local ethics committee of the University of Bayreuth. All subjects provided written informed consent, which included the aim and possible risks of the study, and they could terminate the study at any time without further explanations.

### Test Subjects

Thirty healthy male amateur esports players (age 23.1 ± 3.0 years), who spend an average of 12.3 h per week on esports, were tested. The subjects were recruited through a mailing to the students and employees of the University of Bayreuth and through the esports team of the University of Bayreuth. In total, approximately *n* = 16.000 people were reached directly. A power analysis according to Hopkins (Hopkins, 2020) was performed, which led to a minimum population size of *n* = 17. According to Saeidifard et al. (2018) and Amaro-Gahete et al. (2019), the difference in energy expenditure between sitting and standing is around 14%, which is why we calculated the smallest change in energy expenditure required to exclude a purely sedentary activity with +14%. We assumed a typical error of 13.6% based on past studies we have conducted on resting metabolic rate. The number of participants of *n* = 30 is, therefore, sufficient to prove our hypothesis.

The main inclusion criteria were playing the required game (Counter-Strike: Global Offensive or FIFA 20) for at least 10 h per week with interruptions of <1 month in the last year prior to study participation. The study team set this prerequisite to ensure participants were amateur players with a minimum gaming experience and routine level. Additional inclusion criteria required participants to be in good health, of male gender and between 18 and 40 years of age. Exclusion criteria were regular smoking or medical contraindications for participation in the study. In addition, no coffee, alcohol, or performance-enhancing supplements were to be consumed and no sport was to be practiced on the day of the trial. Table 1 provides the anthropometric and (e)sports activity data for all subjects.

**Table 1:** Characteristics of the subjects.

	**Overall (*n* = 30)**	**CS:GO (*n* = 13)**	**FIFA (*n* = 17)**
Age [years]	23.1 ± 3.0	24.1 ± 3.4	22.3 ± 2.4
Height [cm]	180.4 ± 8.6	181.4 ± 9.5	179.7 ± 8.0
Body mass [kg]	78.5 ± 13.3	82.4 ± 17.0	75.5 ± 9.1
Body fat [kg]	15.2 ± 7.8^#^	19.2 ± 9.2	12.1 ± 4.8
Body fat [%]	18.7 ± 7.2^##^	22.5 ± 7.8	15.8 ± 5.2
Visceral fat [cm^2^]	62.4 ± 36.5^##^	82.0 ± 41.2	47.4 ± 24.1
LBM [kg]	63.3 ± 8.4	63.2 ± 10.5	63.4 ± 6.9
Esports [h/week]	12.3 ± 4.5^##^	14.6 ± 5.9	10.5 ± 1.4
Sporting activity [h/week]	4.5 ± 3.0	3.5 ± 3.6	5.1 ± 2.3

*Values are mean ± SD; LBM, lean body mass; visceral fat [cm^2^] expressed as cross-sectional area; significance of differences between groups:*

# *p < 0.05*,

## *p < 0.01*.

### Study Design

The study was held at the sports medicine laboratory of the University of Bayreuth (Institute of Sports Science). It consisted of one gaming session, split into the pre-gaming, gaming, and post-gaming stage. The pre- and post-gaming phases lasted 10 min, the gaming session at least 30 min and consisted either of Counter-Strike: Global Offensive (CS:GO) or FIFA 20 (FIFA). CS:GO (Valve, Bellevue, US) is a tactical, first-person multiplayer shooter and was played either on the subject's personal device or an ASUS ZenBook UX434F (ASUSTeK COMPUTER INC., Taipei, Taiwan). The soccer simulation video game FIFA 20 (Electronic Arts, Redwood City, US) was performed on PlayStation 4 (Sony, Minato, Tokyo, Japan). Initially on the measuring day, an anthropometric measurement of body composition was performed using bioelectrical impedance analysis (BIA, In-Body 720, Biospace Co. Ltd., Seoul, South Korea), for which the participants were instructed to fast for at least 3 h before they arrived at the laboratory, and esports and sporting activity in hours per week were surveyed *via* a written questionnaire. Pre-, during, and post-gaming respiratory parameters, EE, cardiovascular parameters, as well as blood and hormonal parameters, were determined.

### Respiratory Parameters

Respiratory parameters [oxygen uptake ($\overset{\cdot }{\text{V}}$O_2_), carbon dioxide output ($\overset{\cdot }{\text{V}}$CO_2_), respiratory exchange ratio (RER) and ventilation (VE)] were monitored continuously (METALYZER® 3B, CORTEX Biophysik GmbH, Leipzig, Germany) in sitting position starting 10 min before, over the entire course of the game, and until 10 min thereafter. The spirometry data were obtained breath-by-breath and were analyzed for every 5 s. Data were calculated as 5-min intervals and as 0.5-, 1-, and 2-min intervals after the start of the gaming session. When referring to these data, index entries, therefore, contain either “pre,” the minutes in the game (e.g., RER_**min**2_), “post” or the number of minutes after the end of the game (e.g., RER_min+10_). EE was calculated by converting the measured RER values to their caloric equivalent and multiplying this by the corresponding $\overset{\cdot }{\text{V}}$O_2_ values.

### Cardiovascular Parameters

Cardiovascular parameters [heart rate (HR), cardiac output ($\overset{\cdot }{\text{Q}}$) and stroke volume (SV)] were monitored (PhysioFlow Enduro, Manatec Biomedical, Paris, France) continuously in a sitting position starting 10 min before, over the entire course of the game, and until 10 min thereafter. The cardiovascular data were obtained and analyzed simultaneously to the respiratory parameters due to the connected measurement system (MetaSoft® Studio, CORTEX Biophysik GmbH, Leipzig, Germany).

### Saliva and Blood Analytical Procedures

A saliva sample was taken 15 min before and 15 min after the gaming session to determine the cortisol level (Cort). For this purpose, a cotton roll was soaked with saliva in the mouth for 1–2 min (Salivette® Cortisol, SARSTEDT AG & Co., Nümbrecht, Germany). The saliva was then isolated by centrifugation and analyzed using an enzyme-linked immunosorbent assay. Pre-gaming, 5, 10, 20, and 30 min into the gaming phase, as well as 10 min post-gaming capillary blood samples were taken from an hyperemized earlobe. These were used to determine lactate and blood glucose concentrations (BIOSEN S-Line Lab+, EKF-diagnostic GmbH, Barleben, Germany).

### Statistics

All statistical calculations were performed using Graphpad Prism 8.0 (GraphPad Software, US). Data are presented as the mean ± standard deviation (SD). Data were tested for normal distribution *via* Kolmogorov-Smirnov test. All parameters except cortisol were normally distributed. Possible changes over time were checked by an analysis of variance for repeated measurements (one-way ANOVA) with *post-hoc* tests (Bonferroni *post-hoc* test). Differences between groups at identical time points were calculated using unpaired *t*-tests. The significance of differences in Cortisol levels pre- and post-gaming was analyzed with a non-parametric Wilcoxon matched-pairs signed rank test. The significance level was set at *p* < 0.05.

## Results

### Anthropometric Parameters and (e)Sports Habits

CS:GO and FIFA players differed significantly in absolute and relative body fat, visceral fat, and hours of esports activity per week. No significant differences were found in age, height, body mass, lean body mass, and hours of sporting activity per week between CS:GO and FIFA players (Table 1).

### Respiratory Parameters and Energy Expenditure

**Figure 1:**
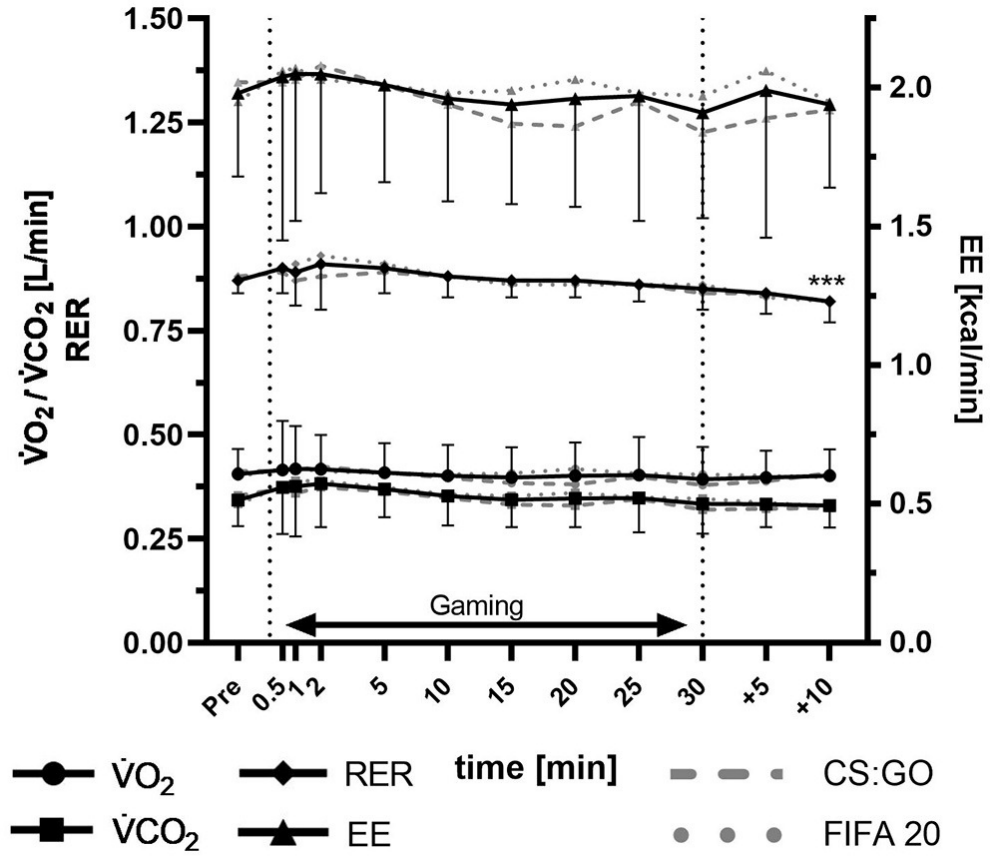
Changes in $\overset{\cdot }{\text{V}}$O2, $\overset{\cdot }{\text{V}}$CO2, RER, and EE before, during and after the gaming session. Significance of differences from baseline (*post-hoc* tests): ^***^p < 0.001.

Figure 1 shows the course in $\overset{\cdot }{\text{V}}$O_2_, $\overset{\cdot }{\text{V}}$CO_2_, RER, and EE before, during, and after the gaming session. $\overset{\cdot }{\text{V}}$O_2_ and $\overset{\cdot }{\text{V}}$CO_2_ remained constant over all three phases. Mean $\overset{\cdot }{\text{V}}$O_2_ during the gaming session was 0.40 ± 0.06 L/min ($\overset{\cdot }{\text{V}}$O_2,pre_: 0.40 ± 0.07 L/min; $\overset{\cdot }{\text{V}}$O_2,post_: 0.40 ± 0.06 L/min). The RER showed a significant time effect (*p* < 0.001) with significant lower values in the post-phase (RER_pre_: 0.87 ± 0.03, RER_min+10_: 0.82 ± 0.05, p_pre;post_ < 0.001). In the calculated EE, no significant changes occurred (EE_pre_: 1.98 ± 0.30 kcal/min; EE_Gaming_: 1.99 ± 0.43 kcal/min; EE_post_: 1.96 ± 0.43 kcal/min). Additionally, there were no significant differences in $\overset{\cdot }{\text{V}}$O_2_, $\overset{\cdot }{\text{V}}$CO_2_, RER, and EE between CS:GO and FIFA players.

### Cardiovascular Parameters

As shown in Table 2, HR and $\overset{\cdot }{\text{Q}}$ showed a significant time effect (*p* < 0.001) and decreased significantly toward the end of the game and the post-phase in relation to HR_min0.5_ and $\overset{\cdot }{\text{Q}}$_min0.5_. In stroke volume, no significant changes occurred. Between CS:GO and FIFA players, no significant differences in HR, $\overset{\cdot }{\text{Q}}$, and SV were detected.

**Table 2:** Heart rate, cardiac output, and stroke volume pre, during and post the gaming session.

**Time [min]**		**Pre**	**0.5**	**1**	**2**	**5**	**10**	**15**	**20**	**25**	**30**	**+5**	**+10**
HR [bpm]	*Overall*	79 ± 10	82 ± 11	81 ± 13	81 ± 12	80 ± 11	78^++^±10	77^++^±10	77^++^±11	78 ± 11	77^+^±10	76^+^±11	74^++^±13
	*CS*:*GO*	82 ± 7	84 ± 6	80^+^±8	81 ± 8	82 ± 7	80 ± 7	79 ± 7	79 ± 7	82 ± 9	80 ± 7	79 ± 8	74 ± 16
	*FIFA*	77 ± 12	81 ± 14	82 ± 16	80 ± 14	78 ± 13	77 ± 13	76^+^±13	76 ± 13	75 ± 12	75 ± 12	74 ± 12	73 ± 11^*^
SV [ml]	*Overall*	95.0 ± 16.4	93.9 ± 20.3	93.2 ± 19.1	96.0 ± 17.5	93.4 ± 16.0	93.7 ± 16.9	95.2 ± 14.1	92.0 ± 19.6	93.7 ± 18.3	92.3 ± 17.8	95.6 ± 16.2	91.3 ± 17.7
	*CS*:*GO*	93.9 ± 20.5	91.5 ± 25.7	90.6 ± 24.8	96.0 ± 21.7	91.6 ± 19.1	93.0 ± 19.8	96.4 ± 13.9	90.0 ± 25.4	92.2 ± 22.3	90.5 ± 21.6	97.8 ± 18.4	88.3 ± 20.5
	*FIFA*	96.0 ± 2.8	95.6 ± 15.7	95.3 ± 13.5	96.0 ± 14.0	94.8 ± 13.5	94.3 ± 14.8	94.2 ± 14.6	93.6 ± 14.0	94.9 ± 14.9	93.7 ± 14.5	94.0 ± 14.7	93.6 ± 15.5
$\overset{\cdot }{\text{Q}}$ [L/min]	*Overall*	7.4 ± 1.2	7.9 ± 1.8	7.7 ± 1.8	7.8 ± 1.6	7.5 ± 1.4	7.4 ± 1.3	7.4 ± 1.1	7.1^++^±1.4	7.3 ± 1.2	7.1^+^±1.3	7.2 ± 1.1	6.8^+^±1.2
	*CS*:*GO*	7.6 ± 1.4	8.0 ± 2.2	7.5 ± 2.0	8.2 ± 1.7	7.8 ± 1.5	7.6 ± 1.4	7.8 ± 0.7	7.3 ± 1.7	7.6 ± 1.2	7.2 ± 1.5	7.7 ± 0.9	6.9 ± 1.3
	*FIFA*	7.3 ± 1.0	7.79 ± 1.5	7.8 ± 1.7	7.6 ± 1.5	7.4 ± 1.3	7.2^++^±1.3	7.2^+^±1.3	7.0 ± 1.2	7.1 ± 1.2	7.0 ± 1.1	6.9 ± 1.2	6.8 ± 1.2

*Values are mean ± SD. HR, heart rate; $\overset{\cdot }{\text{Q}}$, cardiac output; SV, stroke volume. Significance of differences from baseline*:

* *p < 0.05. Significance of differences from t = 0.5*:

+ *p < 0.05*,

++ *p < 0.01*.

### Blood and Hormonal Parameters

Blood glucose showed a significant time effect (*p* < 0.05) with significantly lower values in the post-phase, while lactate remained constant over all three phases (Table 3). Cortisol levels decreased significantly from pre- to post-phase. No significant differences in blood glucose, lactate, and cortisol of CS:GO and FIFA players were found.

**Table 3:** Lactate, glucose, and saliva cortisol pre, during and post the gaming session.

**Time [min]**		**Pre**	**5**	**10**	**20**	**30**	**+10**
Lactate [mmol/L]	Overall	0.9 ± 0.2	0.8 ± 0.2	0.8 ± 0.2	0.8 ± 0.2	0.8 ± 0.2	0.8 ± 0.2
	CS:GO	0.9 ± 0.2	0.8 ± 0.2	0.9 ± 0.2	0.9 ± 0.2	0.8 ± 0.2	0.9 ± 0.2
	FIFA	0.9 ± 0.3	0.9 ± 0.2	0.8 ± 0.2	0.8 ± 0.2	0.8 ± 0.2	0.8 ± 0.3
Glucose [mmol/L]	Overall	5.3 ± 0.8	5.1 ± 0.7	5.1 ± 0.6	5.0 ± 0.7	5.0 ± 0.6	4.9 ± 0.7^*^
	CS:GO	5.3 ± 0.7	5.1 ± 0.5	5.0 ± 0.5	5.0 ± 0.5	5.0 ± 0.5	4.9 ± 0.5
	FIFA	5.2 ± 1.0	5.1 ± 0.8	5.1 ± 0.7	5.0 ± 0.8	5.1 ± 0.7	4.9 ± 0.8
Cortisol [ng/ml]	Overall	3.1 ± 2.9	–	–	–	–	2.2 ± 2.3^**^
	CS:GO	3.6 ± 3.1	–	–	–	–	2.6 ± 2.2
	FIFA	2.6 ± 2.6	–	–	–	–	1.8 ± 2.3^**^

*Values are mean ± SD. Significance of differences from baseline*:

* *p < 0.05*,

** *p < 0.01*.

## Discussion

The present study aims to investigate the extent to which EE and the cardiovascular system are acutely affected in amateur esports players during a 30 min lasting esports unit. The central finding of this study is that a 30 min esports intervention at the amateur level does not affect metabolism and thus EE. Likewise, no stress response is induced in the players. Since very little data exists for amateur players, our results are compared with professional players and casual gamers to enable a relative classification of these performance classes.

### Stress Response

Looking at other sports like chess, which are mentally but not physically demanding, studies found an increased sympathetic activation for professional and amateur players (Troubat et al., 2009; Fuentes-García et al., 2019). Competitive, performance-oriented esports shows very similar tendencies: Looking at professional esports players in a tournament situation, there is an increase in HR and trends toward a heightened $\overset{\cdot }{\text{Q}}$, which implies a high sympathetic activation (Behnke et al., 2019). For example, Rudolf et al. found a slight increase in HR from 102 to 108 bpm, comparing pre to during the tournament situation. This slight increase, as well as the high HR at baseline, are likely caused by the tournament situation and not by the esports activity itself since, in the same players, a relatively low HR (80 bpm) during training of the identical game could be measured (Rudolf et al., 2016). The stress reaction during esports tournaments is partially reinforced at the hormonal level by the significantly elevated cortisol concentrations found in some studies (Schütz, 2016; Schmidt et al., 2020). However, it was not detected in other trials measuring consistent cortisol levels during competitive esports (Oxford et al., 2010; Chaput et al., 2011; Rudolf et al., 2016; Gray et al., 2018). On the other hand, non-competitive casual gaming (CG) was contrary to these results, as no changes in cardiac activity and no hormonal stress indicators could be detected as a reaction to 15–60 min of gaming (Ballard et al., 2006; Arriaga et al., 2008; Staude-Müller et al., 2008; Siervo et al., 2013, 2018).

These findings lead to the expectation that sympathetic activation as an increase in HR, $\overset{\cdot }{\text{Q}}$, SV, and cortisol could also occur in amateur esports, although to a lesser extent than in professional esports. This assumption is also supported by the case study of Haupt et al., in which a mental but no metabolic stress response was triggered in an amateur esports player (Haupt et al., 2021).

In the present study, during the course of the game and just after the end of the game, HR and $\overset{\cdot }{\text{Q}}$ decreased. This suggests that the playing itself did not trigger a stress reaction. At the hormonal level, this assumption is strengthened by the significant decrease in the cortisol level. A rising cortisol level is often used as an indicator for mental stress situations, as it is needed to mobilize glucose for the skeletal musculature to prepare the body for fight-or-flight reactions (Tozman et al., 2017), which does not happen in this study. Similarly, in other competitive, sedentary situations, such as professional chess, no or only small increases in cortisol levels have been found (Tozman et al., 2017; Mendoza et al., 2020). Although the circadian rhythm could have contributed to this, the observed cortisol reduction was significantly more pronounced than what would typically occur during this period in the absence of external influences (Bailey and Heitkemper, 2001).

In physically stressful situations, blood glucose is needed to provide energy for any potential fight-or-flight reactions. During such increased metabolic demands, the release and breakdown of glucose balance each other out, resulting in approximately constant blood glucose levels (Bamberger et al., 1996). In mentally stressful situations, on the other hand, glucose concentration increases due to the elevated glycogenolysis while glucose consumption remains constant. Therefore, the significant decrease in blood glucose values from pre-gaming to 10 min after the end of the game suggests that there is no stress situation caused by playing. The lactate values, which did not change, align with our assumptions because lactate concentration is known to increase during severe stress situations (Hermann et al., 2019).

Overall, when comparing our data to data in the literature, amateur esports should be distinguished in its acute stress-related, physiological effects from professional esports with enormous sympathetic arousal, in which the players find themselves in a highly competitive as well as pressuring situation and are strongly mentally challenged as well as subject to high performance pressure. Therefore, amateur esports can be better compared with the low sympathetic activities in CG.

### Metabolism and Energy Expenditure

In studies on other cognitively oriented sports such as chess, no increased metabolic response could be observed in the professional and amateur levels. However, studies have found an increased stress response in chess players of different performance levels despite the unchanged metabolic response (Troubat et al., 2009; Fuentes-García et al., 2019). At the moment, there is no literature data in the competitive esports area for comparison, but in the CG sector, the same tendencies can be observed in the metabolic reaction, as shown in this study. In chess as well as in CG players, there were no or only minimal increases in respiration rate, $\overset{\cdot }{\text{V}}$O_2_ and EE (Staude-Müller et al., 2008; Lanningham-Foster et al., 2009; Lyons et al., 2011; Barry et al., 2016). As the only study to date that investigated metabolism and EE in esports, the case study by Haupt et al. likewise did not detect an increase in metabolic response or EE. However, a major stress situation occurred during the gaming session (Haupt et al., 2021). At the metabolic level, these findings are supported by this study, as metabolism, expressed by the parameters $\overset{\cdot }{\text{V}}$O_2_, $\overset{\cdot }{\text{V}}$CO_2_, and RER, as well as the EE, showed no acute change due to the 30-min esports intervention.

Overall, the present study's findings suggest that in amateur esports, no acute changes in metabolism or EE occur, while HR and $\overset{\cdot }{\text{Q}}$ slightly decrease. Consequently, the lower HR and $\overset{\cdot }{\text{Q}}$ seem not to be coupled to the metabolic reactions, as it is mentally but not physically induced.

### Games Comparison

Performing esports at an amateur level, players of both games in this study have shown identical physiological responses. However, it is necessary to explicitly distinguish this from the professional area since skill level as well as competition situation could play a major role here and make game-specific investigations necessary.

However, there were differences between the two player groups in body composition and the amount of time spent playing per week. The CS:GO players had a higher body fat percentage than the FIFA players and spent more time playing their respective games per week. Since lean body mass and the exercise activity of both groups did not differ, this could indicate that more gaming hours per week lead to less activity in everyday life. This is supported by a study by DiFrancisco-Donoghue et al. (2020), demonstrating that collegiate esports players are significantly less active than non-esports players and have a higher body fat percentage. However, it is equally conceivable that FIFA as a sports simulation generally attracts players with an already more active lifestyle. All in all, additional research is necessary to make generally applicable statements.

### Practical Implications

The consequence of this would be that esports cannot provide the same health-promoting functions as traditional sports or presumably do not have any health-promoting effects in general. Due to the lack of any form of physical strain, neither the energy metabolism nor the cardiovascular health can be increased, nor can muscles be built, nor can disease-preventing myokines be released (Wilmore, 2003; Chen et al., 2016). Presumably, esports is a form of purely sedentary behavior, which could be associated with potentially harmful effects, such as higher risks for cardiovascular diseases, type 2 diabetes and increased all-cause mortality rates (Katzmarzyk et al., 2019; Saunders et al., 2020). In order to counteract these possible negative health consequences, deliberate physical activity and exercise routines adapted to these demands should be part of the daily life of amateur esports players. Considering the recommendations of the ACSM's guidelines for exercise testing and prescription 2021, adults should perform 150 min of moderate-intensity aerobic physical activity per week for improved health promotion and disease prevention (American College of Sports Medicine, 2021). However, since esports does not constitute aerobic physical activity, these recommendations cannot be fulfilled by esports and additional physical activity is needed to meet these requirements.

### Limitations

Although the power analysis requirements were met, the number of participants was nonetheless limited to *n* = 30, making it difficult to provide generalized conclusions. Additionally, the playing time of 30 min was relatively short and thus may only offer a brief insight into, but not a general overview of the gaming situation. With longer game durations more in line with the daily life of professional or amateur players in esports, prolonged exposure to esports could have different effects on metabolism and stress responses and therefore needs to be investigated. Moreover, only acute effects and not long-term effects were investigated by this study. Furthermore, only two games, CS:GO and FIFA, were examined. The results for other games could differ from those of this study and need to be examined individually. Also, the individual game experience of the players was only partially controlled and may have differed between participants. To determine cardiovascular responses, respectively, sympathetic activity more precisely, blood pressure measurements would have been of the highest interest. However, since conventional blood pressure measurements would have disturbed the game's flow, they were not used in this study.

The regular use of caffeinated or alcoholic products, dietary supplements, or pharmacological agents as well as preliminary fatigue or hydration status were not controlled, which could, to some extent, influence the study outcomes. However, this would probably influence all three measurement phases and keep the changes over the course of the measurement largely unaffected. In addition, changes in emotional state before, during and after the 30-min gaming session were not measured and could have influenced the course of the heart rate.

Additionally, only the weekly sporting activity was surveyed *via* a questionnaire. In order to be able to know the daily physical activity, it would have been necessary to measure the physical activity using an activity tracker. Moreover, it is unclear to what extent these results can be transferred to female amateur players. Further research on gender differences in the psychophysiological response to esports is needed.

## Conclusion

It can be concluded that esports does not have a stimulating effect on EE in amateur players. On the other hand, there is no acute negative stress reaction. However, if several hours a day are regularly spent playing video games or esports, this may lead to the gamer's considerably reduced everyday activity, on the long-term increasing the risk for development of chronic diseases. This tendency is concerning but has not yet been sufficiently investigated and therefore requires further studies to determine the effects of esports on health. It is nevertheless advisable for conscious physical activity and well-adapted exercise routines to be part of the daily routine of amateur esports athletes.

Furthermore, a clear distinction must be drawn between esports professionals and amateurs since professionals are under higher stress influences due to the given tournament or competition situation. Moreover, there could be differences between the physiological responses to different games in the professional area, which has not been investigated yet. Overall, the lack of data is still evident and requires further physiological investigations not only in professional, but, from an epidemiological point of view even more important, also in amateur esports players.

## Data Availability Statement

The original contributions presented in the study are included in the article, further inquiries can be directed to the corresponding author.

## Ethics Statement

The studies involving human participants were reviewed and approved by the Ethics Committee of the University of Bayreuth. The patients/participants provided their written informed consent to participate in this study.

## Author Contributions

RZ, SH, HH, and WS were involved in the conception and design of the study, approved the final version of the manuscript, and agree to be accountable for all aspects of the work in ensuring that questions related to the accuracy or integrity of any part of the work are appropriately investigated and resolved. RZ and SH were involved in the acquisition of data. RZ and WS were involved in the analysis, interpretation of the data, and the drafting of the manuscript. SH and HH were involved in the critical revision of the manuscript. All authors contributed to the study. All authors contributed to the article and approved the submitted version.

## Funding

The study was funded by the regular funds of the University of Bayreuth.

## Conflict of Interest

The authors declare that the research was conducted in the absence of any commercial or financial relationships that could be construed as a potential conflict of interest.

## Publisher's Note

All claims expressed in this article are solely those of the authors and do not necessarily represent those of their affiliated organizations, or those of the publisher, the editors and the reviewers. Any product that may be evaluated in this article, or claim that may be made by its manufacturer, is not guaranteed or endorsed by the publisher.

## References

[B1] Adachi P. J. C. Willoughby T. (2011). The effect of video game competition and violence on aggressive behavior: which characteristic has the greatest influence? Psychol. Viol. 1, 259–274. 10.1037/a0024908

[B2] Amaro-Gahete F. J. Sanchez-Delgado G. Alcantara J. M. A. Martinez-Tellez B. Acosta F. M. Merchan-Ramirez E.. (2019). Energy expenditure differences across lying, sitting, and standing positions in young healthy adults. PLoS ONE 14, e0217029. 10.1371/journal.pone.0217029 31188863 PMC6561541

[B3] American College of Sports Medicine (2021). ACSM's Guidelines for Exercise Testing and Prescription. Liguori G., editor. Philadelphia, PA: Wolters Kluwer.

[B4] Ansgar T. Jannika M. J. (2018). Is eSport a ‘real' sport? Reflections on the spread of virtual competitions. Eur. J. Sport Soc. 15, 311–315. 10.1080/16138171.2018.1559019

[B5] Arriaga P. Esteves F. Carneiro P. Monteiro M. B. (2008). Are the effects of Unreal violent video games pronounced when playing with a virtual reality system? Aggress. Behav. 34, 521–538. 10.1002/ab.20272 18506677

[B6] Bailey S. L. Heitkemper M. M. (2001). Circadian rhythmicity of cortisol and body temperature: morningness-eveningness effects. Chronobiol. Int. 18, 249–261. 10.1081/CBI-100103189 11379665

[B7] Ballard M. E. Hamby R. H. Panee C. D. Nivens E. E. (2006). Repeated exposure to video game play results in decreased blood pressure responding. Media Psychol. 8, 323–341. 10.1207/s1532785xmep0804_1

[B8] Ballard M. E. Wiest J. R. (1996). Mortal kombat (tm): the effects of violent videogame play on males' hostility and cardiovascular responding. J. Appl. Social. Pyschol. 26, 717–730. 10.1111/j.1559-1816.1996.tb02740.x

[B9] Bamberger C. M. Schulte H. M. Chrousos G. P. (1996). Molecular determinants of glucocorticoid receptor function and tissue sensitivity to glucocorticoids. Endocr. Rev. 17, 245–261. 10.1210/edrv-17-3-245 8771358

[B10] Barlett C. P. Anderson C. A. Swing E. L. (2009). Video game effects—confirmed, suspected, and speculative. Simul. Gaming 40, 377–403. 10.1177/1046878108327539

[B11] Barlett C. P. Harris R. J. Bruey C. (2008). The effect of the amount of blood in a violent video game on aggression, hostility, and arousal. J. Exp. Soc. Psychol. 44, 539–546. 10.1016/j.jesp.2007.10.003

[B12] Barry G. Tough D. Sheerin P. Mattinson O. Dawe R. Board E. (2016). Assessing the physiological cost of active videogames (Xbox Kinect) versus sedentary videogames in young healthy males. Games Health J. 5, 68–74. 10.1089/g4h.2015.0036 26625306

[B13] Behnke M. Kosakowski M. Kaczmarek L. (2019). Social challenge and threat predict performance and cardiovascular responses during competitive video gaming. Psychol. Sport Exerc. 46, 101584. 10.1016/j.psychsport.2019.101584

[B14] Chaput J.-P. Visby T. Nyby S. Klingenberg L. Gregersen N. T. Tremblay A.. (2011). Video game playing increases food intake in adolescents: a randomized crossover study. Am. J. Clin. Nutr. 93, 1196–1203. 10.3945/ajcn.110.008680 21490141

[B15] Chen N. Li Q. Liu J. Jia S. (2016). Irisin, an exercise-induced myokine as a metabolic regulator: an updated narrative review. Diabetes Metab. Res. Rev. 32, 51–59. 10.1002/dmrr.2660 25952527

[B16] DiFrancisco-Donoghue J. Werner W. G. Douris P. C. Zwibel H. (2020). Esports players, got muscle? Competitive video game players' physical activity, body fat, bone mineral content, and muscle mass in comparison to matched controls. J Sport Health Sci. S2095-2546(20)30093–4. 10.1016/j.jshs.2020.07.006 32711155 PMC9729923

[B17] Fiore R. Zampaglione D. Murazzi E. Bucchieri F. Cappello F. Fucarino A. (2020). The eSports conundrum: is the sports sciences community ready to face them? A perspective. J. Sports Med. Phys. Fitness 60, 1591–1602. 10.23736/S0022-4707.20.10892-2 32614154

[B18] Fuentes-García J. P. Villafaina S. Collado-Mateo D. La Vega R. de Olivares P. R. Clemente-Suárez V. J. (2019). Differences between high vs. low performance chess players in heart rate variability during chess problems. Front. Psychol. 10, 409. 10.3389/fpsyg.2019.00409 30863351 PMC6400145

[B19] Goodwin J. (2020). On coronavirus lockdown, gamers seek solace and community in video games. USA Today. Available online at: https://eu.usatoday.com/story/tech/2020/04/03/coronavirus-gaming-offers-connection-amid-covid-19-social-distancing/5086472002/

[B20] Gough C. (2021). Leading eSports Countries Ranked by Number of Active eSports Competition Players Worldwide in 2020. Available online at: https://www.statista.com/statistics/780631/esports-competition-country-number-of-players-world/ (accessed January 12, 2022).

[B21] Gray P. B. Vuong J. Zava D. T. McHale T. S. (2018). Testing men's hormone responses to playing league of legends: no changes in testosterone, cortisol, DHEA or androstenedione but decreases in aldosterone. Comput. Human Behav. 83, 230–234. 10.1016/j.chb.2018.02.00415703266

[B22] Haupt S. Wolf A. Heidenreich H. (2021). Energy expenditure during eSports–. Dtsch. Z. Sportmed. 72, 36–40. 10.5960/dzsm.2020.463

[B23] Hedlund D. P. (2021). A typology of eSport players. J. Global Sport Manage. 1–18. 10.1080/24704067.2021.1871858. [Epub ahead of print].

[B24] Hermann R. Lay D. Wahl P. Roth W. T. Petrowski K. (2019). Effects of psychosocial and physical stress on lactate and anxiety levels. Stress 22, 664–669. 10.1080/10253890.2019.1610743 31062999

[B25] Holden J. T. Rodenberg R. M. Kaburakis A. (2017). Esports corruption: gambling, doping, and global governance. Md. J. Int. L. 32, 236. 10.2139/ssrn.2831718

[B26] Hopkins W. G. (2020). Sample-size estimation for various inferential methods. Sportscience 24, 17–27. Available online at: sportsci.org/2020/MBDss.html

[B27] International Esports Federation. (2021). Esports - True Sports? Busan. Available online at: https://iesf.org/esports

[B28] Jagnow H. (2018). ESBD: eSport in Deutschland 2018: Struktur, Herausforderungen und Positionen. Available online at: https://esportbund.de/wp-content/uploads/2019/11/ESBD_eSport_im_Blickpunkt_1-2018-eSport_in-Deutschland_2018.pdf (accessed January 14, 2021).

[B29] Jenny S. E. Manning R. D. Keiper M. C. Olrich T. W. (2017). Virtual (ly) athletes: where eSports fit within the definition of “Sport”. Quest 69, 1–18. 10.1080/00336297.2016.1144517

[B30] Katzmarzyk P. T. Powell K. E. Jakicic J. M. Troiano R. P. Piercy K. Tennant B. (2019). Sedentary behavior and health: update from the 2018 physical activity guidelines advisory committee. Med. Sci. Sports Exerc. 51, 1227–1241. 10.1249/MSS.0000000000001935 31095080 PMC6527341

[B31] Khromov N. Korotin A. Lange A. Stepanov A. Burnaev E. Somov A. (2018). Esports athletes and players: A comparative study. arXiv [Preprint]. arXiv: 1812.03200. Available online at: https://arxiv.org/ftp/arxiv/papers/1812/1812.03200.pdf

[B32] Khromov N. Korotin A. Lange A. Stepanov A. Burnaev E. Somov A. (2019). Esports athletes and players: a comparative study. IEEE Pervasive Comput. 18, 31–39.

[B33] Lanningham-Foster L. Foster R. C. McCrady S. K. Jensen T. B. Mitre N. Levine J. A. (2009). Activity-promoting video games and increased energy expenditure. J. Pediatr. 154, 819–823. 10.1016/j.jpeds.2009.01.009 19324368 PMC2683894

[B34] Lyons E. J. Tate D. F. Ward D. S. Bowling J. M. Ribisl K. M. Kalyararaman S. (2011). Energy expenditure and enjoyment during video game play: differences by game type. Med. Sci. Sports Exerc. 43, 1987–1993. 10.1249/MSS.0b013e318216ebf3 21364477 PMC3271952

[B35] Mendoza G. Jiménez M. García-Romero J. García-Bastida J. Rivilla I. Albornoz-Gil M. C.. (2020). Challenging the top player: a preliminary study on testosterone response to an official chess tournament. Int. J. Environ. Res. Public Health 17, 4–8. 10.3390/ijerph17041204 32069979 PMC7068374

[B36] Newzoo (2018). 2018 Global Esports Market Report. Available online at: https://asociacionempresarialesports.es/wp-content/uploads/newzoo_2018_global_esports_market_report_excerpt.pdf

[B37] Oxford J. Ponzi D. Geary D. C. (2010). Hormonal responses differ when playing violent video games against an ingroup and outgroup. Evolut. Human Behav. 31, 201–209. 10.1016/j.evolhumbehav.2009.07.002

[B38] Reitman J. G. Anderson-Coto M. J. Wu M. Lee J. S. Steinkuehler C. (2020). Esports research: a literature review. Games Cult. 15, 32–50. 10.1177/1555412019840892

[B39] Rudolf K. Grieben C. Achtzehn S. Froböse I. (2016). Stress im eSport - Ein Einblick in Training und Wettkampf. Esport Conference: Professionalisierung einer Subkultur?. Bayreuth.

[B40] Saeidifard F. Medina-Inojosa J. R. Supervia M. Olson T. P. Somers V. K. Erwin P. J.. (2018). Differences of energy expenditure while sitting versus standing: a systematic review and meta-analysis. Eur. J. Prev. Cardiol. 25, 522–538. 10.1177/2047487317752186 29385357

[B41] Saunders T. J. McIsaac T. Douillette K. Gaulton N. Hunter S. Rhodes R. E.. (2020). Sedentary behaviour and health in adults: an overview of systematic reviews. Appl. Physiol. Nutr. Metab. 45(10 Suppl. 2), S197–S217. 10.1139/apnm-2020-0272 33054341

[B42] Schmidt S. C. E. Gnam J.-P. Kopf M. Rathgeber T. Woll A. (2020). The influence of cortisol, flow, and anxiety on performance in e-sports: a field study. Biomed. Res. Int. 2020, 9651245. 10.1155/2020/9651245 32076623 PMC7008303

[B43] Schütz M. (2016). Sportwissenschaftler: eSports-Profis sind wahre Athleten. Available online at: http://www.dw.com com/de/sportwissenschaftler-esportsprofis-sind-wahre-athleten/a*-*19011581 (accessed september 02, 2017).

[B44] Siervo M. Gan J. Fewtrell M. S. Cortina-Borja M. Wells J. C. K. (2018). Acute effects of video-game playing versus television viewing on stress markers and food intake in overweight and obese young men: a randomised controlled trial. Appetite 120, 100–108. 10.1016/j.appet.2017.08.018 28843974

[B45] Siervo M. Sabatini S. Fewtrell M. S. Wells J. C. K. (2013). Acute effects of violent video-game playing on blood pressure and appetite perception in normal-weight young men: a randomized controlled trial. Eur. J. Clin. Nutr. 67, 1322–1324. 10.1038/ejcn.2013.180 24084510

[B46] Statista Research Department (2021). Available online at: https://www.statista.com/forecasts/1277728/physical-or-digital-core-gamers-in-the-us (accessed January 12, 2022).

[B47] Staude-Müller F. Bliesener T. Luthman S. (2008). Hostile and hardened? An experimental study on (de)sensitization to violence and suffering through playing video games. Swiss J. Psychol. 67, 41–50. 10.1024/1421-0185.67.1.41

[B48] Tozman T. Zhang Y. Y. Vollmeyer R. (2017). Inverted U-shaped function between flow and cortisol release during chess play. J. Happiness Stud. 18, 247–268. 10.1007/s10902-016-9726-0

[B49] Troubat N. Fargeas-Gluck M.-A. Tulppo M. Dugué B. (2009). The stress of chess players as a model to study the effects of psychological stimuli on physiological responses: an example of substrate oxidation and heart rate variability in man. Eur. J. Appl. Physiol. 105, 343–349. 10.1007/s00421-008-0908-2 18987876

[B50] Wilmore J. H. (2003). Aerobic exercise and endurance: improving fitness for health benefits. Phys. Sportsmed. 31, 45–51. 10.3810/psm.2003.05.367 20086470

[B51] World Medical Association (2013). WMA Declaration of Helsinki - Ethical Principles for Medical Research involving Human Subjects. Fortaleza: 64th WMA General Assembly. p. 1–7. Available online at: https://www.wma.net/policies-post/wma-declaration-of-helsinki-ethical-principles-for-medical-research-involving-human-subjects/

[B52] Yeo M. Lim S. Yoon G. (2017). Analysis of biosignals during immersion in computer games. J. Med. Syst. 42, 3. 10.1007/s10916-017-0860-y 29159698

[B53] Zwibel H. DiFrancisco-Donoghue J. DeFeo A. Yao S. (2019). An osteopathic physician's approach to the Esports athlete. J. Osteopathic Med. 119, 756–762. 10.7556/jaoa.2019.125 31657829

